# Dengue shock

**DOI:** 10.4103/0974-2700.76835

**Published:** 2011

**Authors:** Senaka Rajapakse

**Affiliations:** Department of Clinical Medicine, Faculty of Medicine, University of Colombo, Sri Lanka

**Keywords:** Dengue, shock, DHF, DSS, myocarditis, corticosteroids, fluids

## Abstract

Shock syndrome is a dangerous complication of dengue infection and is associated with high mortality. Severe dengue occurs as a result of secondary infection with a different virus serotype. Increased vascular permeability, together with myocardial dysfunction and dehydration, contribute to the development of shock, with resultant multiorgan failure. The onset of shock in dengue can be dramatic, and its progression relentless. The pathogenesis of shock in dengue is complex. It is known that endothelial dysfunction induced by cytokines and chemical mediators occurs. Diagnosis is largely clinical and is supported by serology and identification of viral material in blood. No specific methods are available to predict outcome and progression. Careful fluid management and supportive therapy is the mainstay of management. Corticosteroids and intravenous immunoglobulins are of no proven benefit. No specific therapy has been shown to be effective in improving survival.

## INTRODUCTION

Infection with dengue virus imperils about 20 million people every year in tropical and subtropical countries.[1] The mortality rate is around 1–2%. The spectrum of disease manifestations is wide, ranging from asymptomatic or mild infection, through varying degrees of thrombocytopenia and vascular leakage that is typical of dengue hemorrhagic fever (DHF), to a severe shock syndrome and multiorgan failure.[2] Multiple organs can be affected: liver damage, rhabdomyolysis, myocardial depression, and various neurologic and ophthalmologic manifestations have been reported.[2] The case fatality of severe dengue in Asian countries is around 0.5–3.5%.[3]

## CASE REPORT

A 49-year-old woman was admitted to hospital with fever for 5 days. She had no significant previous medical history. Her main symptoms were body aches, headache, loss of appetite, vomiting, and high fever. On day 5 of the illness, a full blood count showed a platelet count of 45,000 mm^3.^ She was admitted to the ICU. On admission, she was conscious and alert, but was restless and looked ill. She had a diffuse cutaneous blanching erythema. Her pulse rate was 120 beats per minute and blood pressure 130/80mmHg, with a postural drop of 20 mmHg. The heart sounds were normal. Her respiratory rate was 28/min, and her lungs were clear. Her abdomen was soft, with no free fluid; epigastric and right hypochondrial tenderness was present. She was neurologically normal. Her electrocardiogram (ECG) was normal, apart from sinus tachycardia. DHF was the likely clinical diagnosis. Intravenous (IV) Hartmann solution 2 ml/kg/h was commenced in view of the postural drop in blood pressure. She became hypotensive 4 h after admission, with blood pressure falling to 70/40 mmHg, and her heart rate increased to 140/min. A repeat ECG showed diffuse T wave inversions. An urgent echocardiogram showed global hypokinesia, with an ejection fraction of 40%. Based on a clinical diagnosis of dengue shock syndrome (DSS) plus myocarditis, dobutamine and noradrenaline infusions were started. Repeat platelet count was 22,000mm^3^ and the hematocrit 48%. Fluids were given with caution, and fresh frozen plasma (FFP) and platelet transfusion was commenced. She then had coffee grounds aspirate through the nasogastric tube and was therefore started on omeprazole 80 mg bolus followed by 8 mg/h infusion. She remained in intractable shock, with no response to inotropes or intravenous hydrocortisone, and required ventilation due to worsening pulmonary edema. Her ECG now showed widespread T wave inversions with first-degree heart block. She progressively deteriorated, became anuric, and developed adult respiratory distress syndrome (ARDS). She also developed complete heart block, for which a temporary pacemaker was inserted. Although rate control was achieved, her blood pressure remained low on maximum inotropes. She was started on IV immunoglobulins 0.4 mg/kg by infusion. Her condition continued to deteriorate, and blood pressure became unrecordable. External cardiac massage alone seemed to raise the blood pressure to recordable levels. Repeat echocardiogram showed a dilated, globally hypokinetic heart, and it appeared that the myocardium was not responding to inotropes at all. She died in asystole shortly afterwards, 15 h after admission. Investigation results received after the patient’s death showed positive IgM and IgG antibodies to dengue. Dengue PCR was also positive. All bacterial cultures were negative. Serum cortisol levels were normal.

## PATHOGENESIS OF SHOCK IN DSS

Dengue viruses are transmitted to humans by infected mosquitoes, mainly *Aedes aegypti and Aedes albopictus*.[2] There are four serotypes of the dengue virus: types 1, 2, 3, and 4. Although referred to as serotypes, these have actually been identified as four different species belonging to the family Flaviviridae and genus Flavivirus. Infection with the dengue virus has a wide range of manifestations. Many infections are asymptomatic. Symptomatic dengue results in two defined syndromes: dengue fever (DF) and DHF/DSS. While DF is a simple, self-limiting febrile illness, DHF is a severe and potentially life-threatening condition. DHF/DSS is characterized by thrombocytopenia, with the resultant hemorrhagic manifestations; in addition, there is increased vascular permeability, resulting in depleted intravascular volume and shock. Severe, profound shock is known to occur in extreme cases and is associated with high mortality.

Two theories have been proposed to explain the pathophysiology of DHF/DSS.[4] According to one theory, DHF/DSS is caused by more virulent strains of the dengue virus. The other theory suggests that DHF/DSS results from abnormal and exaggerated host immune responses – in particular, the production of dengue virus cross-reactive antibodies – which augments the infection. In primary infection with the dengue virus, cross-reactive antibodies that lack neutralizing activity are produced. During secondary infection by a different serotype, the dengue virus and non-neutralizing antibodies form virus–antibody complexes. The Fc portion of these antibodies bind to FcγRI- and FcγRII-bearing cells, resulting in an increased number of cells being infected by the dengue virus.[5] This phenomenon is known as antibody-dependent enhancement and is believed to play an important part in the pathogenesis of shock.[5–8]

Patients with severe dengue die of progressively worsening shock and multiorgan failure. The exact mechanism of this phenomenon is not fully understood although it is thought that increased vascular permeability occurs largely due to malfunction of vascular endothelial cells induced by cytokines or chemical mediators,[9] as also occurs in severe sepsis. It appears that a Th1 response occurs in the first few days of dengue infection; this later switches over to a Th2 response, which correlates with the development of shock.[10] TNF-α, interleukin (IL)-2, IL-6, and IFN-γ levels are highest in the first 3 days of illness, whereas IL-10, IL-5, and IL-4 tend to appear later.[10] IL-2 and IFN-γ are Th1-type cytokines, while IL-5 and IL-4 are Th2-type cytokines. Dengue virus–infected monocytes and endothelial cells have been shown to produce multiple cytokines, including TNF-α.[11,12] In the presence of enhancing antibodies, monocytes infected with dengue virus produce TNF-α. TNF-α has been shown to induce plasma leakage *in vitro*.[13] Basophils and mast cells infected with dengue virus produce IL-1 and IL-6, while IFN-γ, IL-2, and TNF-α are also produced by virus-specific T lymphocytes upon activation.[12,14] Lymphocytes infected with the dengue virus produce IFN-α and IFN-γ,[15] IFN-α levels are higher in patients with DHF, though there is no difference in its levels in different grades of DHF.[16] IFN-γ levels are no different in patients with DF and DHF.[15] The precise role played by interferon in the pathogenesis of dengue shock is unclear. The cytokines TNF-α,[17] IL-6,[10,18,19] IL-8,[19] IL-13,[10,20] IL-18,[10,20] and cytotoxic factor[10] are significantly elevated in DHF as compared with DF. Levels of IL-13,[10] IL-18,[10] and IL-8[21] correlate positively with increasing grades of DHF. On the other hand, IL-12 levels are high in uncomplicated DF, but low in dengue-induced shock.[20] Levels of transforming growth factor-β (an inhibitor of Th1- and enhancer of Th2-type cytokines) correlate with severity of disease and show an inverse relationship with IL-12 levels. Complement activation is a constant finding in DHF. Higher complement levels correlate with increasing disease severity.[4,22] The dengue virus is also known to infect endothelial cells and cause direct damage through apoptosis;[23] nonetheless, it is believed that endothelial activation occurs predominantly through an indirect mechanism.[13] Increased endothelial cell expression of VCAM-1 and ICAM-1 occurs, and TNF-α is a key intermediary in this process.[13] Dengue infections are associated with reduced numbers of CD4+ T-cells, CD8+ T-cells, and natural killer cells.[24] Levels of these cells are lowest at the point when the fever settles and the onset of shock takes place, and increase subsequently. B-cell numbers are not affected.

## CLINICAL FEATURES OF DENGUE ILLNESS PROGRESSING TO SHOCK

Dengue infection should be considered when patients who live in areas where dengue is prevalent present with a febrile illness together with hemorrhagic manifestations or features of shock. The WHO case definitions for dengue shock are shown in Box 1. Dengue infection begins as a febrile illness; the fever is accompanied by constitutional symptoms and a characteristic flushing of the skin. Intermittent high-grade fever accompanied by chills and rigors is a feature. Vomiting, headache, myalgia, epigastric discomfort, and abdominal pain are common, and patients often feel quite ill.[25] The fever lasts 2–7 days and is followed by a fall in temperature; complications of dengue often take place at this point. Patients who remain ill, despite their temperature returning to normal, are more likely to develop shock. Shock generally occurs on day 3–4 of the illness.[26] Thrombocytopenia is a characteristic finding. Platelet counts below 100,000/mm^3^ together with a rise in the hematocrit define DHF. In the classical shock syndrome, increased vascular permeability results in third space fluid loss, leading to pleural effusions, pericardial effusions, ascites, non-cardiogenic pulmonary edema and, subsequently, hypotension. Right hypochondrial pain occurs similar to that seen in cholecystitis; acalculous cholecystitis is a characteristic feature of DHF.[27] Myocarditis is a well-known complication[28] and although often mild it can result in heart block and can be severe enough to result in progressive and intractable acute heart failure with global hypokinesia and acute cardiac dilatation.[28–30] Lactic acidosis, which occurs[31] as a result of the sluggish circulation, possibly contributes to myocardial depression in severe cases. Acute hepatic derangement can occur though fulminant liver failure is rare.[4] Acute renal failure is usually secondary to hypotension in shock syndrome and is associated with increased mortality.[32] Death is usually due to severe hemorrhage or intractable shock with multiorgan failure.

**Box 1: WHO case definitions for dengue shock[1]**

**Features of dengue hemorrhagic fever**

- Fever, or history of acute fever, lasting 2–7 days, occasionally biphasic
- Hemorrhagic tendencies, evidenced by at least one of the following
  - A positive tourniquet test
  - Petechiae, ecchymoses, or purpura
  - Bleeding from the mucosa, gastrointestinal tract, injection sites, or other locations
  - Hematemesis or melena
- Thrombocytopenia (100000/mm3 or less)
- Evidence of plasma leakage due to increased vascular permeability, manifested by at least one of the following;
  - A rise in hematocrit ≥20% above the average for age, sex and population
  - A drop in hematocrit following volume replacement equal to or greater than 20% of the baseline
  - Signs of plasma leakage such as pleural effusion, ascites or hypoproteinemia

**All four of the above PLUS evidence of circulatory failure, manifested by**

- Rapid weak pulse, and
- Narrow pulse pressure (<20mmHg) OR
- Hypotension for age, and
- Cold, clammy skin, and restlessness

Several hemodynamic factors probably contribute to the intractable shock seen in severe dengue. Clearly, the syndrome of increased vascular permeability, the pathophysiology of which is described above, is the prime reason for shock. Classically, an increase in systemic vascular resistance as a result of extravasation of plasma occurs, with a resultant reduction in preload. In addition, associated myocardial depression is likely to contribute to the shock.[28,30] Dehydration due to the associated vomiting and poor intake may also play a role.

## RISK FACTORS FOR THE DEVELOPMENT OF DENGUE SHOCK

The factors which place patients at higher risk of developing dengue shock are not clearly identified yet. DHF/DSS is more likely to occur in infants[33] and the elderly.[33–35] Dengue infection also appears to be more severe in females.[36] Severe dengue is more likely to occur in patients with chronic illness such as diabetes mellitus or asthma.[37,38] Although malnutrition predisposes to many infectious diseases it does not appear to increase the likelihood of severe dengue.[39] The serotype of the infecting virus may influence the severity of dengue; DEN-1 infection, followed by DEN-2 infection, has been reported to be associated with worse outcome.[40] There is some evidence that genetic susceptibility (ethnic variation,[41] HLA typing,[42] etc.) may play a role in the development of dengue shock, but this has not been studied thoroughly.

## DIAGNOSIS

The WHO guidelines on dengue have clearly defined criteria for the diagnosis of dengue shock [Box 1].[1] Confirmation of dengue infection is by serology or detection of dengue viral material in blood by RT-PCR. Dengue-specific IgG and IgM ELISA is widely used.[43] The test is relatively inexpensive, and becomes positive for IgM antibodies on or after day 5 of the fever. IgM ELISA has a sensitivity of 83.9–98.4% and a specificity of 100%.[44] The presence of IgG antibodies indicates previous infection; hence, the presence of both IgG and IgM antibodies suggest the possibility of a secondary infection, although this has not been validated in clinical studies. Cordeiro *et al*.[45] proposed a two-dimensional classifier to differentiate primary and secondary dengue infection based on dengue IgM ELISA and the number of days since the onset of symptoms; the method showed over 90% specificity and sensitivity. Serotyping can also be done using ELISA.[46] RT-PCR for dengue viral material can help to diagnose the illness early, before antibodies become positive; the method, although relatively expensive, is very sensitive and allows for serotyping.[47] While these tests are used for diagnosis of dengue infection, they do not accurately predict which patients are likely to develop dengue shock. The association between high antibody titers or high viral load and the clinical manifestations of dengue has not been studied. No other biochemical investigations are available to predict which patients will develop shock, which is largely a clinical diagnosis. Hemoconcentration and dropping platelet counts herald the onset of shock. Extravasation of fluid due to vascular leakage can be detected radiologically (chest radiography for pleural effusions, echocardiography for pericardial effusions, ultrasonography for ascites). The presence of fluid around the gall bladder, together with thickening of the gallbladder wall, has been shown to be associated with shock.[27,48] None of these features, however, predict the development of severe shock syndrome or mortality. Patients with shock do, however, show a variety of metabolic derangements, including lactic acidosis, elevated transaminases, and rising serum creatinine and blood urea. Pulse oximetry and arterial blood gas analysis showing hypoxia may indicate the development of pulmonary edema, which may be cardiogenic or non-cardiogenic. Electrocardiography is useful in identifying early myocarditis (T wave and ST segment changes are seen).[28] Echocardiography is the main investigation used to diagnose myocardial dysfunction and should be done early when impending shock is suspected. The place of cardiac troponins in diagnosing myocarditis has not been evaluated.

## MANAGEMENT

The WHO has issued guidelines for the management of DSS.[1] Much of the evidence on therapeutic measures in dengue are from children, and evidence from adults is lacking [Table 1]. Close monitoring is required as shock can develop rapidly, and transfer to an ICU is indicated. The patient should be kept under close observation. Pulse, blood pressure, and respiration should be monitored–continuously if possible or at least every 15 min. Oxygen saturation should be monitored using a pulse oximeter, and oxygen should be given by face mask. Two wide-bore cannulae should be inserted for venous access. Blood should be drawn for grouping and cross-matching, blood urea, serum electrolytes, liver function tests, full blood count, prothrombin time, and c-reactive protein. Paracetamol may be used to control the fever.

**Table 1:** Evidence base for key interventions in dengue shock

Therapy	Recommendation
Ideal dose of fluids	Not studied in trials. Intravenous bolus of 10–20 ml/kg recommended by WHO guidelines[1]
Type of fluid	No difference between colloids and crystalloids.[49–51] No evidence from adult studies.
Platelet transfusion	No clear evidence from trials. Required in the presence of hemorrhage. Effect of platelet transfusion short-lived in shock.[52]
Corticosteroids	No clear evidence of benefit.[56–63] Most trials underpowered, poor methodological quality; not studied in adults.
IV immunoglobulins	No evidence of benefit in published literature.
Inotropes and vasopressors	No evidence from clinical trials. Empiric use of vasopressors (dopamine, noradrenaline) in shock, with addition of inotropic agents (dobutamine, adrenaline) if myocardial depression present.
Carbazochrome sodium sulfonate (AC-17)	No evidence of benefit. Single trial, underpowered.[78]
Nasal continuous positive airway pressure (NCPAP)	Effective in acute respiratory failure in DSS.[79]

The only known effective treatment in DSS is timely and aggressive fluid resuscitation. No trials have been conducted comparing the use of intravenous fluids *vs* placebo due to obvious ethical considerations. Fluids used for volume expansion include normal saline, Ringer lactate, 5% glucose diluted 1:2 or 1:1 in normal saline, plasma, plasma substitutes, or 5% albumin. There is no evidence that colloids are superior to crystalloids for resuscitation. Three studies conducted in Vietnam have compared the use of crystalloids and colloids. Dung *et al*.[49] compared four IV fluid regimens (Ringer lactate, normal saline, 3% gelatin, and dextran 70) in 50 children aged 5–15 years with dengue shock; no difference was seen in the occurrence or duration of shock between the groups. No difference was seen between the fluid requirements of crystalloids or colloids. All patients recovered. However, this study was thought to be underpowered to detect a difference between the two groups. Ngo *et al*.[50] conducted a larger study comparing the same fluid regimens in 230 children aged 1–15 years. The study included a larger proportion of patients with more severe grades of DHF. Although a trend towards benefit with colloids over crystalloids was shown, a clear difference between the four regimens was not demonstrated. Subgroup analysis showed that more severely ill patients may benefit from early administration of colloids. Wills *et al*.[51] compared three fluid regimens (Ringer lactate, dextran 70, and 6% hydroxyethyl starch) in 512 children aged 2–15 years with dengue shock. The authors stratified the study population into two groups; moderate shock (pulse pressure >10 and <20 mmHg) and severe shock (pulse pressure <10 mmHg). Patients with moderate shock (*n* = 383) were randomized to receive Ringer lactate, dextran, or starch and those in severe shock (*n* = 129) were randomized to receive dextran or starch. No statistically significant differences were seen in either severity group in the requirement for colloid subsequent to the initial episode of shock, in the volumes of rescue colloid, in total parenteral fluid administered, or in the number of days in the hospital. The authors concluded that treatment with colloids did not provide any benefit over treatment with Ringer lactate in patients with moderate shock. In patients with severe shock, no clear benefit with either starch or dextran was demonstrated. Despite the fact that there is no evidence to support the use of colloids in patients with severe shock, the authors felt that it would be unethical to compare colloids to crystalloids in such patients since it is generally accepted that colloids are needed in cases of severe shock.

The ideal dose of fluids has not been studied in clinical trials, and recommendations are based on practices in centers that have treated large numbers of cases. In the case of shock, fluids should be administered as a rapid (over less than 20 min) intravenous bolus of 10–20 ml/kg body weight. If shock persists, and the hematocrit is rising, plasma, plasma substitutes, or albumin should be given as a rapid bolus and repeated if necessary to a total dose or 20–30 ml/kg of colloid. If shock persists, and particularly if the hematocrit decreases, fresh whole-blood transfusion may be required (10 ml/kg). With appropriate use of fluid resuscitation in DSS, mortality rates have been shown to be <0.2%. It is important to reduce the IV fluids once the patient is recovering, as overhydration can result in intravascular fluid overload once the vascular permeability reverses with recovery.

Platelet transfusions are usually given to patients who develop serious hemorrhagic manifestations or have very low platelet counts, although the exact platelet count at which platelets should be given has not been defined. Transfused platelets survive only for a very short period in patients with shock syndrome.[52] The degree of elevation of circulating platelets after transfusion varies directly with the amount of platelets transfused and inversely with the degree of shock. Blood transfusion is required in patients with severe hemorrhage. There is some evidence of benefit with fresh frozen plasma transfusion in increasing the platelet counts,[53] although the effect of plasma transfusion in dengue shock has not been studied in a controlled clinical trial.

The WHO guidelines for the management of dengue do not discuss the role of corticosteroids. While corticosteroids have various immunosuppressant effects, evidence of beneficial effects of corticosteroids on the deranged immunological mechanisms in dengue is very limited. In patients with ARDS, high-dose corticosteroids have been shown to reduce the levels of the cytokines TNF-α, interleukin (IL)-1β, IL-6, and IL-8.[55] However, Medin *et al*.[54] demonstrated that no reduction was seen in IL-8 after treatment with dexamethasone in patients with dengue. No other studies have examined the effects of corticosteroids on the cytokine cascade. Clinical trials of corticosteroids have been inconclusive so far and for the most part have been underpowered and lacking in methodological quality.[56–63] Some of the early studies demonstrated possible beneficial effects of corticosteroids in dengue shock. Min *et al*.,[56] in a randomized controlled study of children with DSS treated with hydrocortisone, demonstrated a statistically significant mortality benefit with corticosteroids in children aged 8 years and over, although this benefit was not seen in younger children. Futrakul *et al*.[57] reported a series of 22 children with shock syndrome who were treated with pulsed methylprednisolone therapy *vs* saline and plasma replacement. Nine out of 11 children in the corticosteroid-treated group survived, while in the group treated with saline and plasma replacement, all died. Significant hemodynamic improvement was seen in the nine survivors after administration of methylprednisolone. This study was non-blinded and non-randomized. However, subsequent studies of corticosteroids in dengue have all failed to show any benefit either in terms of survival or hemodynamic improvement,[58–62] and a Cochrane review on the subject concluded that there was no evidence of benefit in using corticosteroids in DSS.[64] It must be noted that the previous studies have been small: the total number of patients in all the randomized controlled studies was 284. Of three other non-randomized studies, one study showed no benefit, one study showed a survival benefit, and one very small study showed hemodynamic improvement, including apparent improvement in plasma leakage. All these studies were underpowered, were conducted a long time ago, and have only studied children. There is no evidence from clinical trials regarding the effect of corticosteroids in adults.

Replacement doses of corticosteroids are thought to improve mortality and duration of shock in patients with septic shock who showed a blunted adrenocortical response to the ACTH stimulation test.[65] Cortisol levels are low in a subgroup of patients with septic shock, and a blunted cortisol response to ACTH stimulation is associated with poor prognosis.[66] In contrast, cortisol levels are high in DHF during both the acute and convalescent phases.[67] A correlation between cortisol levels and prognosis in dengue has not been studied. Although some clinicians use steroids in treatment,[68] there is currently no clear evidence to justify the use of corticosteroids in the treatment of DSS. There is a clear need for adequately powered, randomized, double-blind, placebo-controlled clinical trials in both children and adults to fully evaluate the possible benefit or lack of benefit of corticosteroids in dengue infection.

Similar to corticosteroids, the place of IV immunoglobulins (IVIG) is also not mentioned in the WHO guidelines on the management of dengue. Theoretically, the immunomodulatory effects of IVIG can be postulated to have effects on the dengue virus–induced cytokine cascade.[69,70] IVIG selectively triggers the production of IL-1 receptor antagonist (IL-1ra),[70] and also prevents the generation of the complement membrane attack complex (C5b-9) and subsequent complement-mediated tissue damage.[69] There is limited evidence that IVIG is beneficial in the treatment of septic shock in neonates,[71] and a meta-analysis has demonstrated an overall reduction in mortality in adults with severe sepsis/septic shock.[72]

Ostranoff *et al*.[73] reported a series of five patients in Brazil with dengue and severe thrombocytopenia who were treated with IVIG (500 mg/kg/ day infusions over 3 h for 5 days). Clinical improvement, together with improvement in platelet count, was seen in these patients. The only published randomized controlled trial investigating the effect of IVIG on thrombocytopenia showed no benefit;[74] IVIG seemed to have no effect on platelet counts. Seriously ill patients with hemorrhage or shock were excluded from that study and hence the possible effects of IVIG on DSS were not studied. One important conclusion was that IVIG was safe, no significant side effects being encountered during the trial. Alejandria[75] discusses an unpublished randomized controlled trial conducted in the Philippines that compared treatment with IVIG *vs* placebo in children with DSS. This study showed a significant mortality reduction with IVIG treatment. Overall, however, there is currently insufficient evidence to make any recommendation regarding the use of IVIG in dengue shock.[76]

Carbazochrome sodium sulfonate (AC-17) is a hemostatic drug with a capillary-stabilizing action. It has been shown to reduce the vascular hyperpermeability induced by vasoactive substances through an agonist-induced inhibition of phosphoinositide hydrolysis.[77] Its effect in DSS has been investigated in a randomized clinical trial (RCT), conducted in 95 Thai children.[78] The primary outcome measure was the prevention of capillary leakage as evidenced by the presence of pleural effusion, and the secondary outcome was the prevention of shock. No evidence of benefit in either outcome measures was seen with treatment of DSS with AC-17, although the study was underpowered to detect a potential treatment benefit.

An RCT compared the use of nasal continuous positive airway pressure (NCPAP) *vs* oxygen by mask in patients with DSS and acute respiratory failure.[79] The study was conducted in 37 Vietnamese children. The primary outcome measure was a PaO_2_>80 mmHg after 30 min. Although the study was small, NCPAP effectively decreased hypoxemia and reduced the number of children requiring intubation and ventilation. Thus, NCPAP appears to be an effective treatment in acute respiratory failure associated with DSS.

The role of different inotropic and vasopressor agents in dengue shock has not been investigated in clinical trials. Vasopressor drugs such as noradrenaline and dopamine are indicated in shock that is unresponsive to fluids but no clinical trials are available on their use in dengue. In the case of cardiac dysfunction, it is appropriate to use cardiac inotropic drugs such as dobutamine or adrenaline in combination with a vasopressor although, again, no evidence is available.

## CONCLUSION

DSS is a dangerous condition that can rapidly progress to death. Diagnosis is based largely on clinical grounds. No specific therapy has yet been proven to be of value, and the mainstay of management continues to be careful fluid resuscitation. While no definite benefit has been shown of colloids over crystalloids, colloids will continue to have a place in the management of severe shock, pending further research. The role of corticosteroids and immunoglobulins in dengue shock is clearly an area for future research. Much of the recommendations are based on research done in children, and management protocols for adults are based on extrapolation of these findings. Further research on the management of dengue shock in adults is clearly needed.
